# Clinical Application of a Real-Time Telepathology System for Frozen Section Diagnosis in Comparison With Optical Microscope

**DOI:** 10.3389/fmed.2019.00215

**Published:** 2019-10-04

**Authors:** Yu Ting Huang, Salvatore Lorenzo Renne, Mauro Sollai, Domenico Damiani, Paola Bossi, Emanuela Morenghi, Massimo Roncalli, Luca Di Tommaso

**Affiliations:** ^1^Pathology, Humanitas Clinical and Research Center – IRCCS, Rozzano, Italy; ^2^Biostatistic Units, Humanitas Clinical and Research Center – IRCCS, Rozzano, Italy; ^3^Department of Biomedical Sciences, Humanitas University, Rozzano, Italy

**Keywords:** telepathology, e-pathology, real-time, microscope, frozen section

## Abstract

**Background:** The imbalance between the increasing demand of highly specialized service and the reduction of specialists able to release this service is a global challenge for Pathology. This situation applies also to the setting of intra-operatory diagnostic: here the broad presence of Surgical divisions contrasts with the contraction of Pathology departments, progressively concentrated in few hospitals. The use of e-pathology device, such as remote-control microscopes, offers a possible solution to this imbalance.

**Aim:** To prove the non-inferiority of function of a remote-control, real-time microscope named Nano-Eye Device (NED) with the optical microscope (OM) for intra-operatory histological diagnosis.

**Methods:** The study was designed into two phases: discovery and validation. During the discovery phase features influencing the process of adaptation to NED were investigated in detail, focusing on the turnaround time (TAT). Validation phase investigated the diagnostic concordance between NED and OM; as well as sensitivity, specificity, and accuracy of NED in intra-operatory histological diagnosis.

**Results:** During the discovery phase 250 cases were examined. TAT of NED was longer than that of OM (112 ± 89.8 vs. 36 ± 37.9 s) and influenced by the difficulty of the specimen, age of pathologist and the type of the specimen. In the validation phase (185 cases) TAT of NED reduced significantly to 92 ± 86.3 s (*p*: 0.01). NED showed a concordance rate of 98% with OM; the sensitivity (95.65%), specificity (100%), and diagnostic accuracy (98.87%) of NED were equal to that of OM. NED failed to work in 6% during the discovery phase and 4% in the validation.

**Conclusions:** Taken as a whole, the functionality of NED is comparable to OM. It can be the alternative choice for hospital lacking on-site pathology services and one of the tool of e-pathology.

## Introduction

A recent study showed an overall decrease of 17.5% among US pathologists in the period from 2007 to 2017 (1). The same study shed light on the increase of new cancer cases diagnosed per pathologist which raised from 92.81 (2007) to 131.54 (2017). Data from US also suggest that the pathologist workforce demand would rise by 16% in few years because of the additional support needed with an aging population and associated illnesses (2, 3). These trends will likely prompt the onset of a new form of pathology services, based on a widespread use of software systems, with classical procedures and instruments progressively substituted by electronic devices^1^.

Telepathology is the electronic transmission of pathological images, usually derived from microscopes, from one location to another, for the purpose of interpretation and diagnosis. According to the Weinstein Telepathology System Classification, these tools are classified as static, dynamic, hybrid, and using virtual slide (4). Static telepathology allows pathologists the examination of pre-captured still digital images (snapshots) transmitted via e-mail or stored on a shared server. The advantages are relatively low cost, simple technology needed, and low maintenance; the disadvantages include the need for expertise to select the appropriate areas, the limited fields of view and the absence of remote-control access (5, 6). Dynamic telepathology enables pathologists to view entire slide by using robotic, remote-controlled microscopy (7). Advantages include user control of the microscope, good image quality, and fast driving speed; disadvantages are represented by quite expensive technology, the need for integrated software for both host and recipient, high bandwidth requirements, and the need for ongoing technical support and maintenance.

Whole slide imaging (WSI) telepathology involves digitalization of glass slides to produce high-resolution images. Although scanning at higher magnification takes longer and results in larger image files, the digital images have a better resolution and offer superior zoom capability, being suitable for telepathology (8, 9). The rapid diagnosis made with intraoperative evaluation of frozen sections (FS) may cause significant difference in patient care. Although telepathology has not been set for primary FS diagnosis, the interest in its use is growing due to the centralization of pathology services and increasing sub-specialization (10). Dynamic telepathology has been used to diagnose FS by hospitals without a resident pathologist. In this scenario a local microscope, with the sections and maintenance of equipment being provided by local technical staff is governed by a distant pathologist. The diagnostic accuracy of the diagnoses rendered using dynamic telepathology ranged between 89 and 100% (10). Static telepathology systems are inappropriate to deliver this type of service since founded on the presence of an expert (a pathologist) to capture the representative image while WSI may provide an alternative way^2^.

In order to further investigate the functionality of dynamic telepathology for the diagnosis of frozen section, the goal of this study is to compare the accuracy and effectiveness of a real-time system microscope named Nano-Eye Device for Digital Pathology (briefly NED) with the conventional optical microscope.

## Materials and Methods

### Study Design

The study was performed in the Department of Pathology of Humanitas Clinical and Research Center—IRCCS, Rozzano, Italy, between January and June 2019. Five pathologists were recruited to participate in the study. In the following description, we referred to them as pathologist A to E. All frozen sections seen by any of these five pathologists during their intra-operatory examination turn were considered for the study. The study was designed into two phases: discovery phase (January 2019—April 2019) and validation phase (May 2019—June 2019). This study was approved by the Ethic Committee of Humanitas Clinical and Research Center (ID 2178/2019).

### Discovery Phase

During the discovery phase the pathologist in charge of frozen section duty was requested to reach a diagnosis using a classical optical microscope (OM). After the diagnosis had been released to the surgeon, the same frozen section was mounted on the slide holder and inserted into NED, and then evaluated by the same pathologist working from the computer in a remote-controlled way. A detailed description of NED and its functions is reported in the Supplementary Material. The following variables were recorded: quality of image; turnaround time (TAT); difficulty of the specimen; concordance between diagnosis; major and minor problems encountered. The definition of each variable is detailed in the Supplementary Material.

*Aim of the discovery phase* was to get familiar with all the possible aspects of NED such as looking at a screen and not in the oculars, moving the slide with a mouse and not using micrometrics, etc., and compared them with those, well-known, of an OM.

### Validation Phase

A validation study should closely emulate the real-world clinical environment in which the technology will be used (11). In keeping with this, once completed the 4 months formation period warranted by the discovery phase, the pathologists involved in the study were requested to reach a diagnosis of frozen section using NED. In line with the discovery phase, during the validation phase we recorded the pathologist who used NED, the diagnosis released, TAT employed and the difficulty of the specimen. In particular, the diagnoses were classified as positive (margin involved; malignant tumor present; specimen adequate for diagnostic purpose), negative (margin free; benign/no tumor on the specimen; specimen not adequate for diagnostic purpose) or deferred to stable section. Immediately after the pathologist in charge of frozen section duty (P1) reached the diagnosis on NED, the same frozen section was assigned to a second pathologist (P2) involved in the study but blind to the original diagnosis. P2 was requested to reach a diagnosis using the same diagnostic categories (positive, negative or deferred to stable section) but working with OM. If P1 and P2 agreed on the diagnosis, this was considered as conclusive and communicated to the surgeon. In cases of discordance, the original slide was immediately discussed by P1, P2 and an expert pathologist (>20 years of activity) to reach a conclusive diagnosis which was then communicated to the surgeon. The diagnostic classification adopted, allowed the evaluation of the concordance rate between OM and NED diagnosis, specificity, sensitivity, diagnostic accuracy. Cases inadequate to reach a diagnosis using NED were diagnosed using OM.

*Aim of the validation phase* was to evaluate if the performance of NED, in terms of concordance and TAT, was comparable to that of OM in the routine activity of a Pathology department.

### Statistics

Data were described as number and percentage, or mean ± standard deviation and range, as appropriated. Sensitivity, specificity and accuracy were calculated respect the conclusive diagnosis (reached by P1 and confirmed by P2 or, in case of disagreement between P1 and P2, that reached by P1 and P2 under the supervision of P3). Difference in TAT for NED in the two phases was evaluated with Wilcoxon test. Variations in TAT for NED and OM among different pathologist were explored with Kruskal Wallis test. Differences in TAT for NED and OM between easy and difficult specimen were explored with Mann Whitney test. A *p* < 0.05 was considered as significative. All analyses were made with Stata15.

## Results

### Discovery Phase

**Figure 1:**
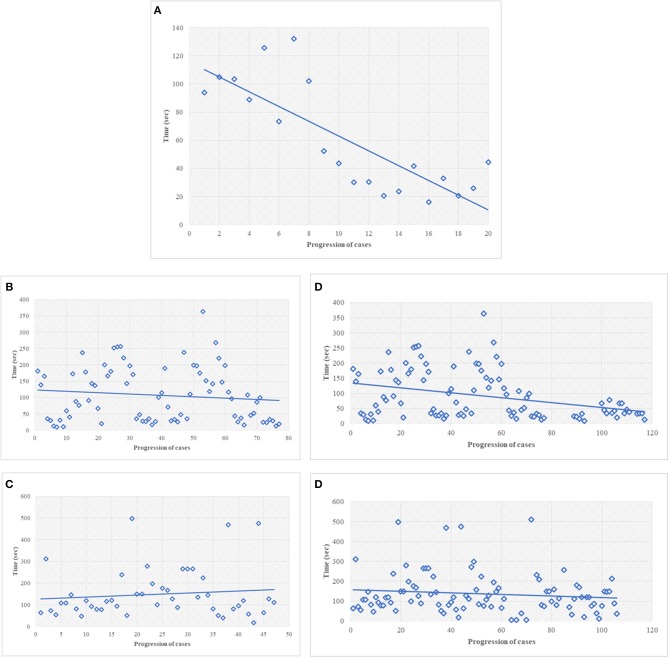
**(A)** Discovery phase, turn-around-time performance of the most common type of specimen, i.e., prostatic margin, using NED. In this figure, every single dot represents the mean TAT of each time of use, considering all the pathologists involved. The first dot represents the mean of the TAT of all five pathologists the first time they saw a prostate specimen; the last dot represents the mean of the TAT of the pathologists (two) the 20th time they saw at a prostate specimen. All the pathologists looked to prostatic specimen at least 10 times (the 10th dot shows the mean of TAT of five pathologists). **(B,C)** Discovery phase, the best **(B)** and worst **(C)** turn-around-time performance for all type of cases, using NED. In this figure and in the followings **(D,E)** every dot represent a single case seen by a single pathologist. **(D)** Turn-around-time performance at the end of the study, same pathologist shown in **(B)**. **(E)** Turn-around-time performance at the end of the study, same pathologist shown in **(C)**.

The discovery phase encompassed 250 cases, covering a broad spectrum of organs, as shown in Table 1. Among this series, 15 cases (6%) were inadequate to reach a diagnosis due to NED shut down. The remaining cases allowed a conclusive diagnosis being either adequate (190 cases, 76%) or limited (45 cases, 18%). These latter cases (n° 235, 94%) permitted the comparison between OM and NED. Taking into consideration them, the mean TAT used to reach the diagnosis was 36 ± 37.9 s using OM (range 4–345 s) and 112 ± 89.8 s using NED (range 6–497 s), with a ratio NED/OM of 3.11. The chronological age of pathologist and the degree of difficulty of the specimen under investigation impacted on the process of adjustment to NED. Older pathologists, despite their longer working experience, showed higher mean TAT time with NED but not with OM, as shown in Table 2. Difficult cases (n° 44) were diagnosed in 163.1 ± 111 s as compared to 100.8 ± 80 s for easy cases (n° 191). The type but not the number of cases evaluated influenced the habituation to NED. Taking into consideration the most frequent specimen seen (prostatic margin, 32%), all five pathologists showed a clear-cut decrease in TAT using NED, as shown in Figure 1A. Focusing on the number of cases diagnosed, five pathologists showed different trends; in particular, those two pathologists concluding the higher number of diagnosis had opposite results, as shown in Figures 1B,C.

**Table 1:** Distribution of cases by body site, discovery phase.

Appendix−1 (<1%) Biliary tract−13 (~5%) Bladder−4 (~1%) Breast−14 (~6%) Bronchus−10 (4%)	Esophagus−2 (<1%) Eye−2 (<1%) Lung−7 (~3%) Lymph node−54 (~22%) Nose−2 (<1%) Omentum−1 (<1%)	Pancreas−14 (~6%) Pericardium−2 (<1%) Peritoneum−5 (2%) Pleura−3 (~1%) Prostate−81 (~32%)	Soft tissue−8 (~3%) Stomach−2 (<1%) Testicle−2 (<1%) Ureter−11 (~4%) Uterus and ovary−10 (4%) Vagina−2 (<1%)

**Table 2:** TAT of OM and NED by chronological age of pathologists, discovery phase.

	**Pathologist**				
	**A** **(50–55 yrs)**	**B** **(45–50 yrs)**	**C** **(35–40 yrs)**	**D** **(30–35 yrs)**	**E** **(30–35 yrs)**
OM (sec)	27.8 ± 15.8	69.3 ± 66	21.3 ± 16.8	34.8 ± 19.2	33.1 ± 21.6
NED (sec)	167.2 ± 97.2	149.2 ± 111.8	74.2 ± 53.9	68.8 ± 46.7	106.9 ± 82.3

*A to E, pathologists involved in the study; TAT, turn around time; yrs, years; OM, optical microscope; NED, nano eye device; sec, seconds*.

### Validation Phase

A total of 185 cases were recorded in validation phase and detailed in Table 3. They were adequate, limited or inadequate to reach a diagnosis in, respectively, 160 (87%), 17 (9%), and 8 (4%). Inadequate and limited cases decreased, from 6 to 4% and from 18 to 13%, respectively, from the discovery to the validation phase.

**Table 3:** Distribution of cases by body site, validation phase.

Breast−13 (~7%) Bronchus−4 (~3%) Eye−6 (~3%) Lung−6 (~3%) Lymph node−59 (~32%) Omentum−4 (~2%)	Pancreas−10 (~5%) Pericardium−4 (~2%) Peritoneum−5 (~3%) Pleura−2 (~1%) Prostate−34 (~18%) Soft tissue−8 (~4%)	Tongue−4 (~2%) Ureter−12 (~6%) Urethra−2 (~1%) Uterus and ovary−4 (~2%) Vessels−4 (~2%) Vocal cord−4 (~2%)

Among 177 cases suitable to the diagnosis with NED, 173 had the same diagnosis (concordance rate 98%) and 4 (2%) showed a discrepancy when reviewed with OM. Discordant cases were referred to a senior pathologist for arbitration. After this review, there was a conclusive agreement to consider incorrect two cases diagnosed with OM and two diagnosed with NED. Accordingly, NED and OM showed the same values of sensitivity 95.65%, specificity 100%, and diagnostic accuracy 98.87%.

During the validation phase, the mean TAT recorded with NED decreased from 112 ± 89.8 s to 92 ± 86.3 s (*p* = 0.01). Besides this general decrease, a reduction of TAT was observed also in specific settings. TAT for both difficult and easy cases decreased compared to those in the discovery phase. The percentage of reduction is more for easy cases (19%) than for difficult cases (12%), as shown in Table 4. All the pathologists, except one, showed a decrease of TAT, being the direct correlation with chronological age retained, as shown in Table 5. Finally, as shown in Figures 1D,E, also the number of cases, when they overwhelmed 80 cases, impact on the process of adaptation to NED.

**Table 4:** TAT of NED by degree of difficulty, comparison between the discovery and the validation phases.

	**TAT NED (sec) discovery phase**	**TAT NED (sec) validation phase**
Difficult specimen	163.1 ± 111 (n° 44 cases)	143 ± 105.5 (n° 36 cases)
Easy specimen	100.8 ± 80 (n° 191 cases)	81.6 ± 78.4 (n° 141 cases)

*sec, seconds*.

**Table 5:** TAT of NED by chronological age of pathologists, comparison between the discovery and the validation phases.

	**Pathologist**				
	**A** **(50–55 yrs)**	**B** **(45–50 yrs)**	**C** **(35–40 yrs)**	**D** **(30–35 yrs)**	**E** **(30–35 yrs)**
Discovery phase (sec)	167.2 ± 97.2	149.2 ± 111.8	74.2 ± 53.9	68.8 ± 46.7	106.9 ± 82.3
Validation phase (sec)	106.9 ± 35.3	126 ± 90	102 ± 144.4	68.4 ± 56	38.2 ± 19

*A to E, pathologists involved in the study; yrs, years; sec, seconds*.

## Discussion

NED is a classical microscope (objectives, etc.) integrated by a computer system warranting all the numerous possibilities of an electronic device (such as a mobile phone warrants the use of several app), including the live control by a distant position. Due to this last characteristic it represents a possible solution for the management of intra-operatory diagnostic in hospitals performing surgical procedures but lacking on-site pathology service. This study compared the effectiveness of NED to that of optical microscope (OM) in the process of intra-operatory histological diagnosis.

The urgency of time is the most relevant characteristic of intra-operatory diagnostic and one of the main pressures for pathologists. Therefore, our first goal was to identify variable influencing adaptation to NED. As a first point, it was observed a strict correlation between the chronological age of pathologists and the turn-around-time (TAT) used to reach the diagnosis with NED, with older pathologists—those with longer experience- taking more time than younger. This aspect was observed during the discovery and confirmed in the validation phase although the mean time of TAT recorded by every pathologist decreased in the validation as a clear effect of the learning process. The most probable explanation to this observation, related to NED but not to OM, is that the fluency of using a new technique tool is likely age-related and not mitigated by professional experience. Another aspect influencing the time to reach a conclusive diagnosis was the degree of difficulty of cases, with TAT of easier cases decreasing more than that of difficult cases (19 and 12%, respectively) as the experience with NED increased. The distinction between easy and difficult cases, despite poorly reproducible, raised a further aspect to be considered. In the study phase 16% of prostatic specimens (13/81) and sentinel lymph-nodes (9/54), the most common specimens sent to FS, respectively, for sparring nerve procedure and diagnosis of metastatic foci, were considered as difficult. In the validation phase these values dropped to 5% (2/34) and 3% (3/59). These data suggest that the confidence with a new device, such as NED, may also influence the subjective evaluation to the difficulty of the case. The type and the number of specimens also influenced the adjustment to NED. In particular, it was observed that being exposed to the same type of specimen fastened the process of learning. Indeed, a clear learning curve was seen already during the discovery phase for the most common type of specimen (i.e., prostatic margin). By contrast, taking into consideration the number of specimen (independently from type), a decreasing trend of TAT was reached generally when >80 cases have been evaluated. As a result of progressive adaptation to NED, significant reduction of the overall mean TAT of NED was seen at the end of the validation phase, making it closer to the urgency and pressures of intra-operatory examination.

Considering the clinical relevance of intra-operatory histological examination where different diagnoses may lead to different surgical choices, diagnostic accuracy of NED was the other key point to be evaluated during the study. In the discovery phase, all the diagnoses were concordant between OM and NED (data not shown), but the appropriate washout period of at least 2 weeks requested when a single pathologist is looking at the same slide (11) was missing making the evaluation just a part of the adjustment to NED. In the validation phase the same slide was seen by two different pathologists making the comparison between NED and OM feasible. Once excluded the inadequate cases, NED showed a concordance rate of 98% with OM. Discordant cases where reviewed at a multi-head microscope by the two pathologists involved in the diagnosis lead by a 3rd pathologist and a conclusive diagnosis reached. This approach classified the four discordant cases as due to a mistake made on OM (n° 2) and on NED (n° 2). Accordingly, NED showed the same sensitivity (95.65%), specificity (100%), and diagnostic accuracy (98.87%) of OM.

Even though the accuracy, sensitivity, and specificity of NED are equal with that of OM and the TAT has its potential to be improved, the reliability of NED is still impacted by the major problems (those causing NED shut down) encountered in use. The percentage of success to work was 94% in the discovery phase (6% failure) and raised to 96% in the validation phase (4% failure). This improvement was because of the regular feedback from users and the optimization of the system before the validation phase.

In conclusion, it is undeniable that the combined advantages of NED (e.g., same accuracy to OM; similar TAT with OM; platform to develop artificial intelligence algorithm (12); possible impact on education, secondary consultation, conferencing) provide an alternative choice for the peripheral hospital lacking on-site pathology service and offer better quality of medical care. If NED can create a win-win situation for doctors and patients, then it is a good instrument and will also contribute to the development of e-pathology.

## Data Availability Statement

The datasets generated for this study are available on request to the corresponding author.

## Ethics Statement

The studies involving human participants were reviewed and approved by Ethic Committee of Humanitas Clinical and Research Center (ID 2178/2019). The patients/participants provided their written informed consent to participate in this study.

## Author Contributions

YH designed and managed the study, organized the database, and wrote the draft of the manuscript. SR participated in the study and drafted the statistical analysis. MS, DD, and PB participated in the study. EM supervised the statistical analysis. MR edited the manuscript. LD designed and supervised the study and edited the manuscript. All authors contributed to manuscript revision, read, and approved the submitted version.

### Conflict of Interest

The study was sponsored by a NTP fund. The authors declare that the research was conducted in the absence of any commercial or financial relationships that could be construed as a potential conflict of interest.

## Footnotes

^1^OMICS International. *Digital Pathology and e-Pathology*. OMICS International. Available online at: https://www.omicsonline.org/conferences-list/e-pathology (accessed June 2019).

^2^Lowe J. *Telepathology: Guideline From the Royal College of Pathologists*. The Royal College of Pathologists. Available online at: https://www.rcpath.org/uploads/assets/6b748863-cf1a-4693-970ab7a28fba11c4/telepathology-guidance-from-the-royal-college-of-pathologists.pdf (accessed May 2019).
