# Supplementary material for: Clinical Application of a Real-Time Telepathology System for Frozen Section Diagnosis in Comparison With Optical Microscope
Source: Front Med (Lausanne). 2019 Oct 4;6:215. doi: 10.3389/fmed.2019.00215 (PMC6788327; doi:10.3389/fmed.2019.00215)
Supplement: Supplementary file 1 [file Data_Sheet_1.docx]

**CLINICAL APPLICATION OF A REAL-TIME TELEPATHOLOGY SYSTEM FOR FROZEN SECTION DIAGNOSIS IN COMPARISON WITH OPTICAL MICROSCOPE**

*Yu Ting Huang^1,§^, et al*

**Supplementary Materials and Methods**

*Nano-Eye Device for Digital Pathology (NED)*

NED is a real-time, live view digital pathology system, based on a dynamic/robotic system, composed of host computer and camera mounted on a remote-controlled microscope equipped with 6 objectives (1.25×, 2×, 4×, 10×, 20×, 40×). The specific technical features are listed in Supplementary Table 1.

**Supplementary Table 1**. Specific technical features of the microscope used by NED

| Object | Specific technique |
| --- | --- |
| Optic system | Optic with infinite correction of type UIS2 |
| Illumination | White LED: 18,000 cd/m2 to 35,000 cd/m2; average duration approximately 100,000 hours |
| Objective revolver | Revolver with 6 positions |
| Magnifying objectives | 1.25×; 2×; 4×; 10×; 20×; 40×; optional 60× in substitution of one of the previous |
| Power supply | 230-240 V AC |
| Dimension and weight | Length 31 cm; Height 42 cm; Width 41 cm.; weight 20 kg. |
| Max absorbed power | 250 W |
| Environmental conditions of use | For interior  Ambient temperature 15/40 °C  Voltage fluctuation not to exceed ±10% |

NED allows the observation of slides mounted with coverslip and the coverslip must be presented on the superior part. In the NED-DP 2.0 version the slideholder can handle up to 4 samples and the label must be positioned on the right side with respect to the direction of insertion in the slideholder. The insertion and positioning of the slide are guided by a system with magnets. It is enough to accompany the slide in the track of the motorized trolley. At the end of the sample insertion procedure the system the image will be visible on the monitor.

The control functions of the movement and the optimization of the images (see the scheme below) can be managed entirely by the control panel of the software interface either through keyboard or through the mouse of which the central area is dedicated to displaying the live image.


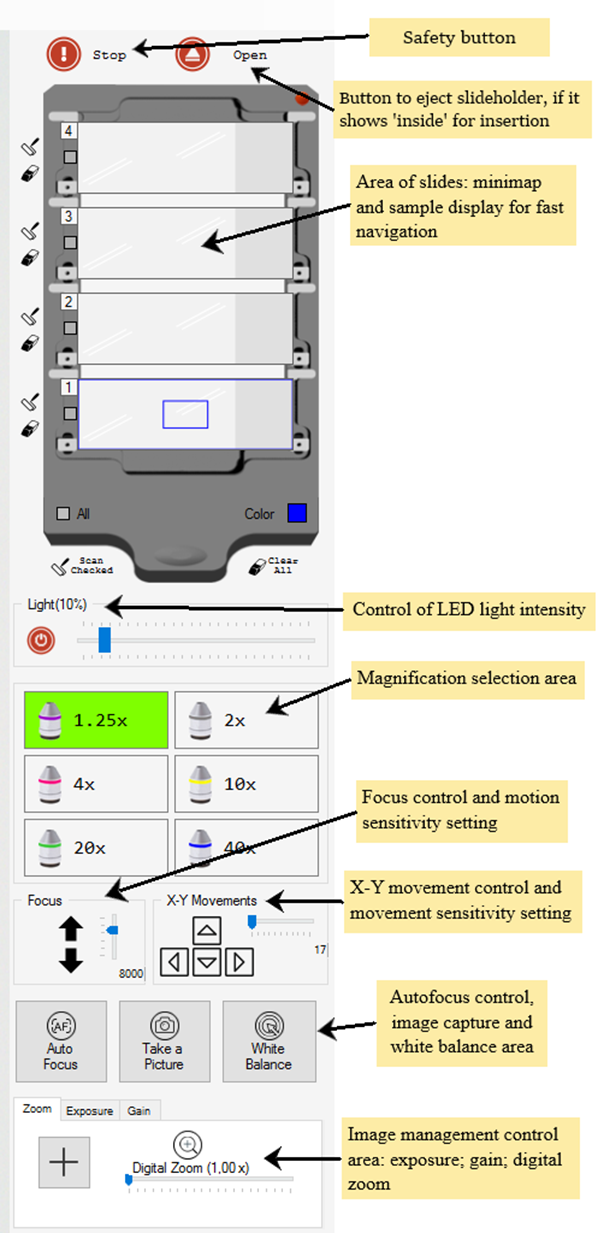


After inserting the sample slide NED creates a minimap as a preview for each individual slide. The blue square as a guide provides synchronized location information. It is possible to select the individual slides even in non-consecutive positions and scan. Once this sample map has been created, you can identify the area being observed with all the available magnifications. By clicking with the mouse inside the minimap, the system will place the selected area on the minimap in the main window. This function can be used at any magnification and for each of the individual slides. The image below shows the histological slide, the minmap and the control function as they appear on the monitor.


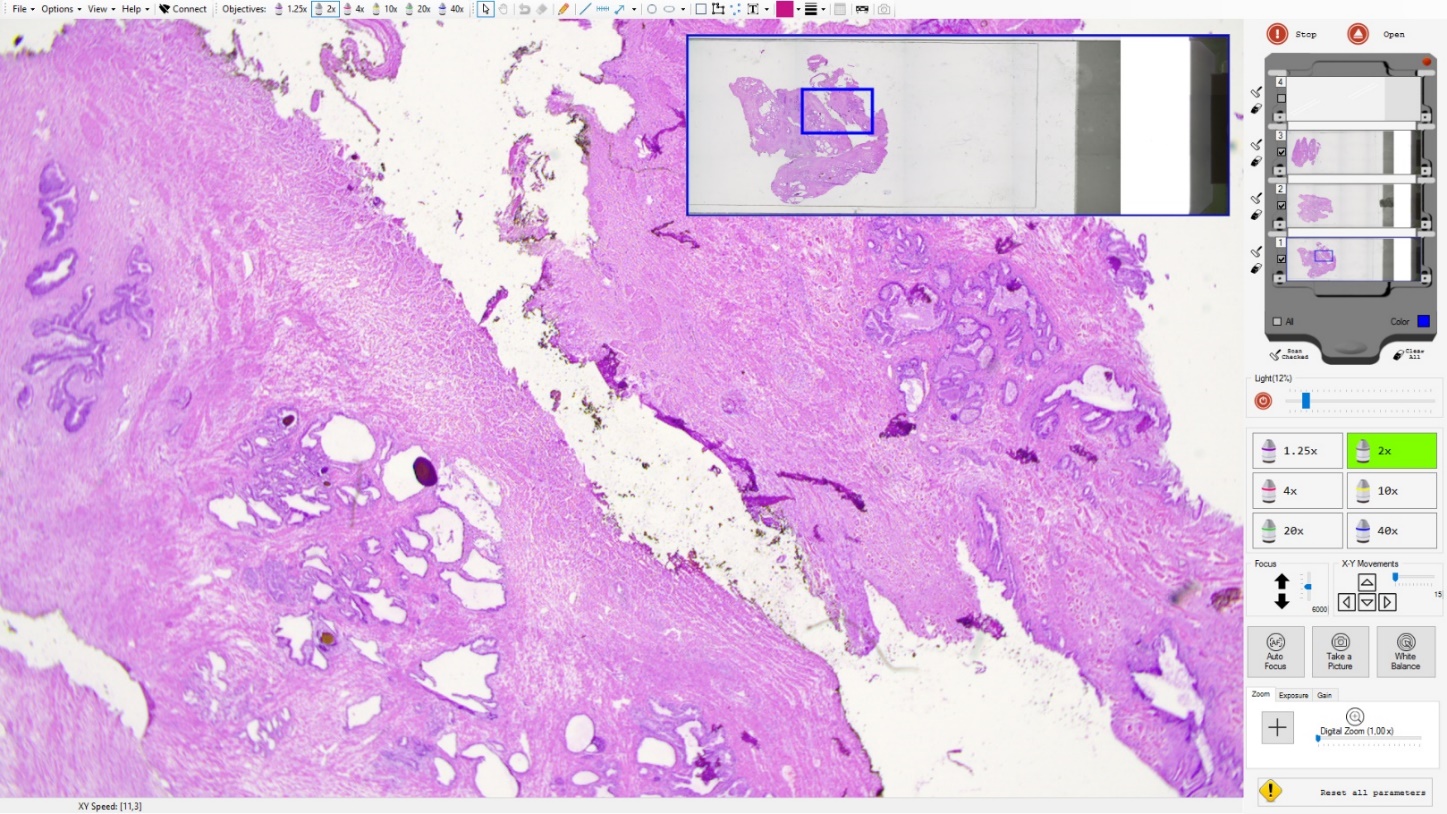


Regarding the X-Y and Z movements (focus), from the control panel, you can set the sensitivity of the X-Y-Z movements. Using the arrows in the focus area, you can move and define the most correct focusing position. With arrows in the movement area you can move the slide on the XY plane to determine the observation area. You can also control the X-Y and Z movements by using the mouse. Holding down the left mouse button and moving, the arrows indicate the direction of movement. Using the mouse wheel, you can act on the focusing of the sample. For each single magnification a minimum displacement value is already set for X-Y and Z axes, which should allow sufficient movement precision. However, it is possible to set new values ​​using cursors. The X-Y position is directly connected to the relative position on the minimap. It is, therefore, possible to interact for the XY shift from the minimap, which generates a shift on the main window in real-time.

The system is calibrated to obtain the best balance of the RGB channels in order to optimize the chromatic result of the image. However, samples with different dominated tones or distribution of objects may not be optimal. Among the various controls that manage image quality such as brightness, contrast, gain, etc., there is a function that simplifies the management of the RGB color aspect. You can perform a quick and precise white calibration by drawing on the image a resizable rectangle in a white area not covered by a sample. This operation will allow the system to calibrate the RGB ratios on the entire sample. On preparations which are particularly complex from the chromatic point of view, the operation could be necessary for the change of magnification.

Each individual lens has been set to the specific nominal focus value. When the lens is changed, the focusing system automatically positions itself with respect to the characteristic value of the selected lens. This prefocus function allows you to have an image already in optimal focus conditions. Different conditions of slide and coverslip samples could affect the operation of the prefocus. Using the autofocus button, it is possible to select two different autofocus modes: One-Shot and Continuous.

- One-Shot: activates the autofocus function only once on the framed area. the XY displacement of the slides could result in a loss of focus caused by the non-perfect flatness of the sample.
- Continuous: Activate the autofocus function and keep it active until it is manually deactivated by the operator. In this case, when the slide XY is moved, the system is able to keep the sample in focus even in the presence of planarity defects.

The autofocus system works by identifying the zones of greatest contrast and defines the position of medium focusing on the framed area. In case of preparations with different thicknesses i.e. cytology, the autofocus system could be positioned in the middle area with respect to the thickness of the sample. Autofocus is also influenced by the depth of field characteristic of each lens as the magnification changes. The autofocus used with low magnification lenses (1.25×, 2×, 4×) may have minor differences in focus. Using autofocus in continuous mode the system could slow down in its calculation and data processing functions. Also, the sample can influence the speed of autofocus execution. Samples that are poorly contrasted or with significant differences in thickness could lead to a longer autofocus processing time.

After the sample insertion, in order to allow the manipulation from the remote station, you have to connect to the network to start by using the connect button at the main station . Once it is connected, the pathologist at remote station can enter the account and using the same function as at main station as we described previously. In this remote-control mode, the control of functions will be transferred to the remote station, while you can still see the real-time image from the main station. If you want to turn off the remote-control mode, you can simply use the disconnect button at the main station and the control of function will be back.


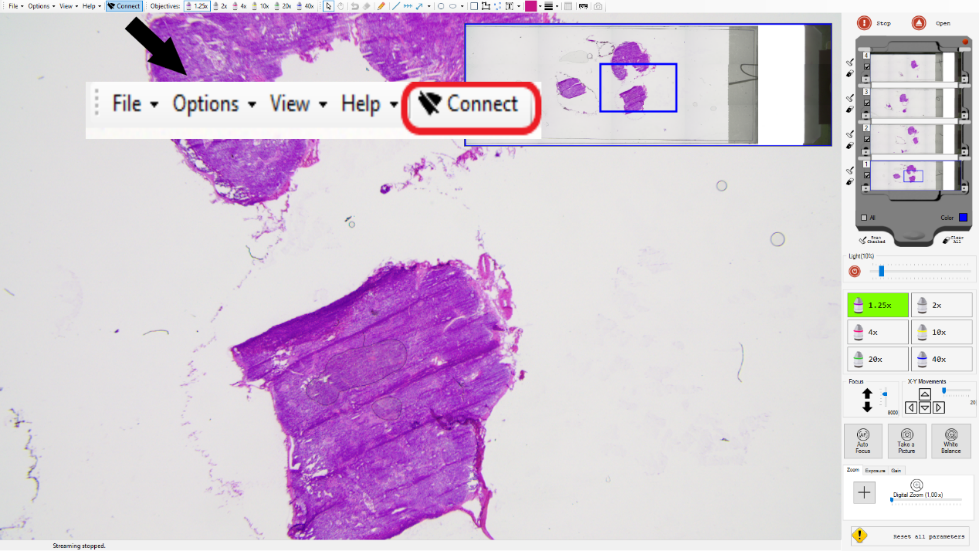


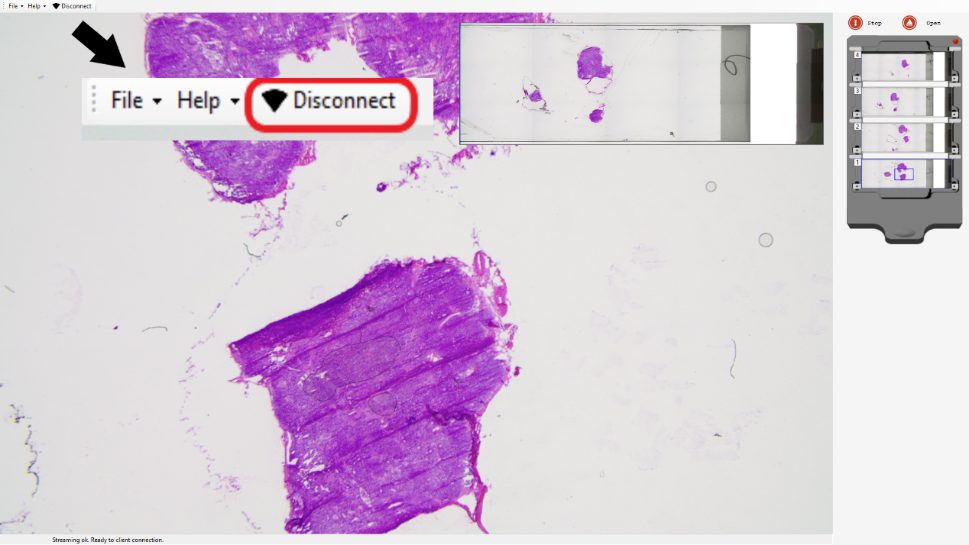


*Criteria evaluated during the study.*

*Quality of NED image*, classified as:

Adequate: suitable to reach the diagnosis; no problem during use.

Limited: sufficient to reach the diagnosis; limited by slowness, blurry image, focus.

Inadequate: insufficient to reach the diagnosis.

*Turn-Around-Time (TAT)*, time requested to complete the procedure, as follows:

OM: seconds elapsing between the moment when the pathologist put the slide (start) and removed it (end) on/from the microscope.

NED: seconds elapsing between the moment when the pathologist started and finished looking at the slide on the monitor.

*Difficulty of the specimen*, classified as easy or difficult, according to pathologist own judgment and according to specific case (e.g. a pancreatic margin with severe chronic pancreatitis was considered as difficult while the same specimen without features of pancreatitis was considered as easy).

*Concordance* between the diagnosis using OM and NED for the same pathologist (i.e. intra-observer variability), as suggested in the guideline for validating WSI [31]:

Concordant: same diagnosis between OM and NED;

Discordant: different diagnoses between OM and NED.

Discordant cases were further divided into:

Minor discrepancy: the two diagnoses had the same therapeutic choice;

Major discrepancy: the two diagnoses had a different therapeutic choice.

*Major and minor problems* encountered during use of NED, defined as follows:

- - Major problems: those caused crash of NED;
  - Minor problems: those caused limited quality of image.
